# Supplementary material for: Expanding the Scope of Adenoviral Vectors by Utilizing Novel Tools for Recombination and Vector Rescue
Source: Viruses. 2024 Apr 23;16(5):658. doi: 10.3390/v16050658 (PMC11125593; doi:10.3390/v16050658)
Supplement: Supplementary file 1 [file viruses-16-00658-s001.zip › viruses-2975046-supplementary.pdf]

viruses

MDPI

Supplementary Materials

Expanding the Scope of Adenoviral Vectors by Utilizing Novel Tools for Recombination and Vector Rescue

Table S1. Plasmid List. List of plasmids used and generated for this study, including general features, size and GenBank Accession Number.

Plasmid Name	Size	Features	Accession
**pGPS1.1**	4814 bp	GPS-1 Genome Priming System transposon donor; kanamycin resistance (KanR); tetracycline resistance (TetR)	10666445
**pKD46**	6329 bp	red-recombineering plasmid; Arabia sugar dependent expression of lambda red phage exo, beta and gam protein; temperature sensitive; β-lactamase expression (AmpR)	10829079
**pKSB2**	6457 bp	Single-copy BACmid backbone; chloramphenicol resistance (CamR)	
**pBWH-A12-RC**	40563 bp	Genomic BACmid; carrying HAdV-A12 genome in reverse orientation; ITRs flanked by ACT sequences; chloramphenicol resistance (CamR)	PP442630
**pBWH-B3**	41781 bp	Genomic BACmid; carrying HAdV-B3 genome; ITRs flanked by ACT sequences; chloramphenicol resistance (CamR)	PP442581
**pBWH-B35-RC**	41232 bp	Genomic BACmid; carrying HAdV-B35 genome in reverse orientation; ITRs flanked by ACT sequences; chloramphenicol resistance (CamR)	PP442594
**pBWH-C2-RC**	42375 bp	Genomic BACmid; carrying HAdV-C2 genome in reverse orientation; ITRs flanked by ACT sequences; chloramphenicol resistance (CamR)	PP442595
**pBWH-F41-RC**	40626 bp	Genomic BACmid; carrying HAdV-F41 genome in reverse orientation; ITRs flanked by ACT sequences; chloramphenicol resistance (CamR)	PP442608
**pBWH-SE25**	42957 bp	Genomic BACmid; carrying SAdV-E25 genome; ITRs flanked by ACT sequences; chloramphenicol resistance (CamR)	PP442609
**pUC-B3-Ins0**	2232 bp	HAdV-B3-targeted transfer vector, designated Insertion site 0; empty multi cloning site; β-lactamase expression (AmpR)	PP442615
**pUC-B3-Ins1**	2625 bp	HAdV-B3-targeted transfer vector, designated Insertion site 1; empty multi cloning site; β-lactamase expression (AmpR)	PP442616
**pUC-B3-Ins2**	3055 bp	HAdV-B3-targeted transfer vector, designated Insertion site 2; empty multi cloning site; β-lactamase expression (AmpR)	PP442617
**pUC-B3-Ins3**	2255 bp	HAdV-B3-targeted transfer vector, designated Insertion site 3; empty multi cloning site; β-lactamase expression (AmpR)	PP442618
**pUC-B3-Ins4**	2414 bp	HAdV-B3-targeted transfer vector, designated Insertion site 4; empty multi cloning site; β-lactamase expression (AmpR)	PP442619
**pUC-E4-Ins0**	2231 bp	HAdV-E4-targeted transfer vector, designated Insertion site 0; empty multi cloning site; β-lactamase expression (AmpR)	PP442625
**pUC-E4-Ins1**	2821 bp	HAdV-E4-targeted transfer vector, designated Insertion site 1; empty multi cloning site; β-lactamase expression (AmpR)	PP4462626
**pUC-E4-Ins2**	2386 bp	HAdV-E4-targeted transfer vector, designated Insertion site 2; empty multi cloning site; β-lactamase expression (AmpR)	PP442627
**pUC-E4-Ins3**	2779 bp	HAdV-E4-targeted transfer vector, designated Insertion site 3; empty multi cloning site; β-lactamase expression (AmpR)	PP442628
**pUC-E4-Ins4**	2134 bp	HAdV-E4-targeted transfer vector, designated Insertion site 4; empty multi cloning site; β-lactamase expression (AmpR)	PP442629

**pUC-B3-I0-mCMV-GFPt2aGLuc**	5014 bp	HAdV-B3-targeted transfer vector, designated Insertion site 0; constitutive expression of T2A-peptide-linked GFP and gaussia luciferase; β-lactamase expression (AmpR)	PP442610
**pUC-B3-I1-mCMV-GFPt2aGLuc**	5407 bp	HAdV-B3-targeted transfer vector, designated Insertion site 1; constitutive expression of T2A-peptide-linked GFP and gaussia luciferase; β-lactamase expression (AmpR)	PP442611
**pUC-B3-I2-mCMV-GFPt2aGLuc**	5837 bp	HAdV-B3-targeted transfer vector, designated Insertion site 2; constitutive expression of T2A-peptide-linked GFP and gaussia luciferase; β-lactamase expression (AmpR)	PP442612
**pUC-B3-I3-mCMV-GFPt2aGLuc**	5037 bp	HAdV-B3-targeted transfer vector, designated Insertion site 3; constitutive expression of T2A-peptide-linked GFP and gaussia luciferase; β-lactamase expression (AmpR)	PP442613
**pUC-B3-I4-mCMV-GFPt2aGLuc**	5196 bp	HAdV-B3-targeted transfer vector, designated Insertion site 4; constitutive expression of T2A-peptide-linked GFP and gaussia luciferase; β-lactamase expression (AmpR)	PP442614
**pUC-E4-I0-mCMV-GFPt2aGLuc**	5013 bp	HAdV-E4-targeted transfer vector, designated Insertion site 0; constitutive expression of T2A-peptide-linked GFP and gaussia luciferase; β-lactamase expression (AmpR)	PP442620
**pUC-E4-I1-mCMV-GFPt2aGLuc**	5603 bp	HAdV-E4-targeted transfer vector, designated Insertion site 1; constitutive expression of T2A-peptide-linked GFP and gaussia luciferase; β-lactamase expression (AmpR)	PP442621
**pUC-E4-I2-mCMV-GFPt2aGLuc**	5168 bp	HAdV-E4-targeted transfer vector, designated Insertion site 2; constitutive expression of T2A-peptide-linked GFP and gaussia luciferase; β-lactamase expression (AmpR)	PP442622
**pUC-E4-I3-mCMV-GFPt2aGLuc**	5561 bp	HAdV-E4-targeted transfer vector, designated Insertion site 3; constitutive expression of T2A-peptide-linked GFP and gaussia luciferase; β-lactamase expression (AmpR)	PP442623
**pUC-E4-I4-mCMV-GFPt2aGLuc**	4916 bp	HAdV-E4-targeted transfer vector, designated Insertion site 4; constitutive expression of T2A-peptide-linked GFP and gaussia luciferase; β-lactamase expression (AmpR)	PP442624
**pBWH-B3delE3-I0n-Kan**	39090 bp	Genomic BACmid; carrying HAdV-B3 genome, E3-region deleted; Insertion Site 0n primed for HFR via insertion of KanR; ITRs flanked by ACT sequences; chloramphenicol resistance (CamR); kanamycin resistance (KanR)	PP442583
**pBWH-B3delE3-I0n-mCMV-GFPt2aGLuc**	40882 bp	Genomic BACmid; carrying HAdV-B3 genome, E3-region deleted; constitutive expression of T2A-peptide-linked GFP and gaussia luciferase from Insertion site 0n; ITRs flanked by ACT sequences; chloramphenicol resistance (CamR); kanamycin resistance (KanR)	PP442584
**pBWH-B3delE3-Ins0-Kan**	39535 bp	Genomic BACmid; carrying HAdV-B3 genome, E3-region deleted; Insertion Site 0 primed for HFR via insertion of KanR; ITRs flanked by ACT sequences; chloramphenicol resistance (CamR); kanamycin resistance (KanR)	PP442589
**pBWH-B3delE3-I0-mCMV-GFPt2aGLuc**	40925 bp	Genomic BACmid; carrying HAdV-B3 genome, E3-region deleted; constitutive expression of T2A-peptide-linked GFP and gaussia luciferase from Insertion site 0; ITRs flanked by ACT sequences; chloramphenicol resistance (CamR); kanamycin resistance (KanR)	PP442582
**pBWH-B3delE3-Ins1-Kan**	39142 bp	Genomic BACmid; carrying HAdV-B3 genome, E3-region deleted; Insertion Site 1 primed for HFR via insertion of KanR; ITRs flanked by ACT sequences; chloramphenicol resistance (CamR); kanamycin resistance (KanR)	PP442590
**pBWH-B3delE3-I1-**	40925 bp	Genomic BACmid; carrying HAdV-B3 genome, E3-region deleted; constitutive expression of T2A-peptide-linked GFP and gaussia luciferase from	PP442585

**mCMV-GFPt2aGLuc**		Insertion site 1; ITRs flanked by ACT sequences; chloramphenicol resistance (CamR); kanamycin resistance (KanR)	
**pBWH-B3delE3-Ins2-Kan**	38712 bp	Genomic BACmid; carrying HAdV-B3 genome, E3-region deleted; Insertion Site 2 primed for HFR via insertion of KanR; ITRs flanked by ACT sequences; chloramphenicol resistance (CamR); kanamycin resistance (KanR)	PP442591
**pBWH-B3delE3-I2-mCMV-GFPt2aGLuc**	40925 bp	Genomic BACmid; carrying HAdV-B3 genome, E3-region deleted; constitutive expression of T2A-peptide-linked GFP and gaussia luciferase from Insertion site 2; ITRs flanked by ACT sequences; chloramphenicol resistance (CamR); kanamycin resistance (KanR)	PP442586
**pBWH-B3delE3-Ins3-Kan**	39512 bp	Genomic BACmid; carrying HAdV-B3 genome, E3-region deleted; Insertion Site 3 primed for HFR via insertion of KanR; ITRs flanked by ACT sequences; chloramphenicol resistance (CamR); kanamycin resistance (KanR)	PP442592
**pBWH-B3delE3-I3-mCMV-GFPt2aGLuc**	40925 bp	Genomic BACmid; carrying HAdV-B3 genome, E3-region deleted; constitutive expression of T2A-peptide-linked GFP and gaussia luciferase from Insertion site 3; ITRs flanked by ACT sequences; chloramphenicol resistance (CamR); kanamycin resistance (KanR)	PP442587
**pBWH-B3delE3-Ins4-Kan**	39353 bp	Genomic BACmid; carrying HAdV-B3 genome, E3-region deleted; Insertion Site 4 primed for HFR via insertion of KanR; ITRs flanked by ACT sequences; chloramphenicol resistance (CamR); kanamycin resistance (KanR)	PP442593
**pBWH-B3delE3-I4-mCMV-GFPt2aGLuc**	40925 bp	Genomic BACmid; carrying HAdV-B3 genome, E3-region deleted; constitutive expression of T2A-peptide-linked GFP and gaussia luciferase from Insertion site 4; ITRs flanked by ACT sequences; chloramphenicol resistance (CamR); kanamycin resistance (KanR)	PP442588
**pBWH-E4delE3-I0n-Kan**	39073 bp	Genomic BACmid; carrying HAdV-E4 genome, E3-region deleted; Insertion Site 0n primed for HFR via insertion of KanR; ITRs flanked by ACT sequences; chloramphenicol resistance (CamR); kanamycin resistance (KanR)	PP442597
**pBWH-E4delE3-I0n-mCMV-GFPt2aGLuc**	40895 bp	Genomic BACmid; carrying HAdV-E4 genome, E3-region deleted; constitutive expression of T2A-peptide-linked GFP and gaussia luciferase from Insertion site 0n; ITRs flanked by ACT sequences; chloramphenicol resistance (CamR); kanamycin resistance (KanR)	PP442598
**pBWH-E4delE3-Ins0-Kan**	39549 bp	Genomic BACmid; carrying HAdV-E4 genome, E3-region deleted; Insertion Site 0 primed for HFR via insertion of KanR; ITRs flanked by ACT sequences; chloramphenicol resistance (CamR); kanamycin resistance (KanR)	PP442603
**pBWH-E4delE3-I0-mCMV-GFPt2aGLuc**	40938 bp	Genomic BACmid; carrying HAdV-E4 genome, E3-region deleted; constitutive expression of T2A-peptide-linked GFP and gaussia luciferase from Insertion site 0; ITRs flanked by ACT sequences; chloramphenicol resistance (CamR); kanamycin resistance (KanR)	PP442596
**pBWH-E4delE3-Ins1-Kan**	38959 bp	Genomic BACmid; carrying HAdV-E4 genome, E3-region deleted; Insertion Site 1 primed for HFR via insertion of KanR; ITRs flanked by ACT sequences; chloramphenicol resistance (CamR); kanamycin resistance (KanR)	PP442604
**pBWH-E4delE3-I1-mCMV-GFPt2aGLuc**	40938 bp	Genomic BACmid; carrying HAdV-E4 genome, E3-region deleted; constitutive expression of T2A-peptide-linked GFP and gaussia luciferase from Insertion site 1; ITRs flanked by ACT sequences; chloramphenicol resistance (CamR); kanamycin resistance (KanR)	PP442599
**pBWH-E4delE3-Ins2-Kan**	39390 bp	Genomic BACmid; carrying HAdV-E4 genome, E3-region deleted; Insertion Site 2 primed for HFR via insertion of KanR; ITRs flanked by ACT sequences; chloramphenicol resistance (CamR); kanamycin resistance (KanR)	PP442605
**pBWH-E4delE3-I2-**	40938 bp	Genomic BACmid; carrying HAdV-E4 genome, E3-region deleted; constitutive expression of T2A-peptide-linked GFP and gaussia luciferase from	PP442600

**mCMV-GFPt2aGLuc**		Insertion site 2; ITRs flanked by ACT sequences; chloramphenicol resistance (CamR); kanamycin resistance (KanR)	
**pBWH-E4delE3-Ins3-Kan**	39001 bp	Genomic BACmid; carrying HAdV-E4 genome, E3-region deleted; Insertion Site 3 primed for HFR via insertion of KanR; ITRs flanked by ACT sequences; chloramphenicol resistance (CamR); kanamycin resistance (KanR)	PP442606
**pBWH-E4delE3-I3-mCMV-GFPt2aGLuc**	40938 bp	Genomic BACmid; carrying HAdV-E4 genome, E3-region deleted; constitutive expression of T2A-peptide-linked GFP and gaussia luciferase from Insertion site 3; ITRs flanked by ACT sequences; chloramphenicol resistance (CamR); kanamycin resistance (KanR)	PP442601
**pBWH-E4delE3-Ins4-Kan**	39646 bp	Genomic BACmid; carrying HAdV-E4 genome, E3-region deleted; Insertion Site 4 primed for HFR via insertion of KanR; ITRs flanked by ACT sequences; chloramphenicol resistance (CamR); kanamycin resistance (KanR)	PP442607
**pBWH-E4delE3-I4-mCMV-GFPt2aGLuc**	40938 bp	Genomic BACmid; carrying HAdV-E4 genome, E3-region deleted; constitutive expression of T2A-peptide-linked GFP and gaussia luciferase from Insertion site 4; ITRs flanked by ACT sequences; chloramphenicol resistance (CamR); kanamycin resistance (KanR)	PP442602

Table S1: Bacmid assembly primers**.** Primers generating ITR overlaps used for genomic bacmid assembly. ITR homologous sequences are underlined.

Virus	Primer name	Sequence
**HAdV-A12**	BWHA12for	AGTCCAGTATAAGGTATATTATTAGATAGGCCTCCGGGGTCCAC-TGCAATTACTTCTCGACCAATTCTCATGTTTGAC
	BWHA12rev	AGTCCAGTATAAGGTATATTATTAGATAGGCCTCCGGGGTCCAC-TGCAATTATAAACTCGACAGCGACACACTTGC
**HAdV-B3**	BWHB03for	GTTGGCACCATTCCATCTATAAGGTATATTATATAGA-TAGCCTCCGGGGTCCACTG-CAATTACTTCTCGACCAATTCTCATGTTTGAC
	BWHB03rev	GTTGGCACCATTCCATCTATAAGGTATATTATATAGA-TAGCCTCCGGGGTCCACTG-CAATTATAAACTCGACAGCGACACACTTGC
**HAdV-B35**	BWHB35for	ATTGGCAC-CATTCCATCTATAAGGTATATTATTGATGATGCCTCCGGGGTCCACTGCAATTACTTCTCGACCAATTCTCATGTTTGAC
	BWHB35rev	ATTGGCAC-CATTCCATCTATAAGGTATATTATTGATGATGCCTCCGGGGTCCACTGCAATTATAAACTCGACAGCGACACACTTGC
**HAdV-C2**	BWHC02for	ATATTGGCTTCAATCCAAAA-TAAGGTATATTATGATGATGCCTCCGGGGTCCACTG-CAATTATAAACTCGACAGCGACACACTTGC
	BWHC02rev	ATATTGGCTTCAATCCAAAA-TAAGGTATATTATGATGATGCCTCCGGGGTCCACTG-CAATTACTTCTCGACCAATTCTCATGTTTGAC
**HAdV-F41**	BWHF41for	TTCCAGCTTTAAGGTATATTATTGATGATGCCTCCGGGGTCCAC-TGCAATTACTTCTCGACCAATTCTCATGTTTGAC
	BWHF41rev	TTCCAGCTTTAAGGTATATTATTGATGATGCCTCCGGGGTCCAC-TGCAATTATAAACTCGACAGCGACACACTTGC
**SAdV-E25**	BWHSE25for	TAACGCGCACAAAAAGTTT-GAGGTATATTATTGATGATGGCCTCCGGGGTCCACTG-CAATTACTTCTCGACCAATTCTCATGTTTGAC
	BWHSE25rev	TAACGCGCACAAAAAGTTTGAGGTATATTATTGAA-GATGGCCTCCGGGGTCCACTG-CAATTATAAACTCGACAGCGACACACTTGC

Table S3. Primers used for cloning of recombinant virus bacmids & transfer vectors.

Name	Sequence
**B3-Ins0_For**	AGTCATTTCGCGGTTATGCCAGG
**B3-Ins0_Rev**	ACCTCCTGTGGCAGGAAGCG
**B3-Ins0-Kan_For**	TTCTGAGTCATTTCGCGGTTATGCCAGGGTGGAGTATTTAAATCCGTGTGGGCG-GACAATAAAGTCTTAAACTGAA
**B3-Ins0-Kan_Rev**	AGATAACCTCCTGTGGCAGGAAGCGCAGGTGTCTCATTTAAATAAATGTGGGCG-GACAAAATAGTTGG
**B3-Ins0n-Kan_for**	AACTGTTGAATGGTAGATTTATGTTTTTTTCTTGCGATTTAAATTCGTGTGGGCG-GACAATAAAGTCTTAAACTGAA
**B3-Ins0n-Kan_rev**	AAACGTATCCCTTGCTGAAGTGAGTTTGGCGGATCCATTTAAATAAATGTGGGCG-GACAAAATAGTTGG
**B3-Ins1_For**	GAGTGGAAGCGCTTCTTTTG
**B3-Ins1_Rev**	CATGCACTCCGACCTTGCTA
**B3-Ins1-Kan_For**	GCCATGAGTGGAAGCGCTTCTTTTGAGGGGGGAGTATTTAAATCCGTGTGGGCG-GACAATAAAGTCTTAAACTGAA
**B3-Ins1-Kan_Rev**	AACACCATGCACTCCGACCTTGCTATGACTGGGTGATTTAAATAAATGTGGGCG-GACAAAATAGTTGG
**B3-Ins2_for**	ACTCTTAAAGAGTAGCCCTTGCCC
**B3-Ins2_rev**	TGTGAACCCGTCAGGGGAAATGAA
**B3-Ins2-Kan_For**	CGCTGACTCTTAAAGAGTAGCCCTTGCCCGCGCTCATTTAAATACGTGTGGGCG-GACAATAAAGTCTTAAACTGAA
**B3-Ins2-Kan_Rev**	CTTTGTGTGAACCCGTCAGGGGAAATGAAACCAGGATTTAAATAAATGTGGGCG-GACAAAATAGTTGG
**B3-Ins3_For**	AACTACTCAGGCAACCCTCA
**B3-Ins3_rev**	TGAAAGCGCTTTAAAAGACTAT
**B3-Ins3-Kan_For**	CCAGAAACTACTCAGGCAACCCTCATAACCTCCCCATTTAAATCCGTGTGGGCG-GACAATAAAGTCTTAAACTGAA
**B3-Ins3-Kan_Rev**	GACTGTGAAAGCGCTTTAAAAGACTATCCCGATGCATTTAAATAAATGTGGGCG-GACAAAATAGTTGG
**B3-Ins4_For**	AAAAGGCACAGGAGAATAAAAAAT
**B3-Ins4_Rev**	ATGGAATGGTGCCAACATGT
**B3-Ins4-Kan_For**	AAAGTAAAAGGCACAGGAGAATAAAAAATATAATTATTTAAATCCGTGTGGGCG-GACAATAAAGTCTTAAACTGAA
**B3-Ins4-Kan_Rev**	TATAGATGGAATGGTGCCAACATGTAAATGAGGTAATTTAAATAAATGTGGGCG-GACAAAATAGTTGG
**E4-Ins0_For**	AGTAATTTAGTGTTTATGACAGGGAG
**E4-Ins0_Rev**	CTCATCGGGCAGGTCTCTTA
**E4-Ins0-Kan_For**	ATCTGAGTAATTTAGTGTTTATGACAGGGAGGAGTATTTAAATTCGTGTGGGCG-GACAATAAAGTCTTAAACTGAA
**E4-Ins0-Kan_Rev**	ATTTCCTCATCGGGCAGGTCTCTTAGGTGCCTCATATTTAAATAAATGTGGGCG-GACAAAATAGTTGG
**E4-Ins0n-Kan_For**	AGGCAAAGAGTAGGGTGACTGGGTGATGACTGGTTTATTTAAATTCGTGTGGGCG-GACAATAAAGTCTTAAACTGAA
**E4-Ins0n-Kan_Rev**	GCTGGTAATCCCTGCTGAAATGGGTTTCTTGACTCCATTTAAATAAATGTGGGCG-GACAAAATAGTTGG
**E4-Ins1_For**	TGGATGTGACGGAGGACCTG
**E4-Ins1_Rev**	CACGCCCTGCGTCCCTGCTA
**E4-Ins1-Kan_For**	GTGTGTGGATGTGACGGAGGACCTGCGACCCGATCATTTAAATGCGTGTGGGCG-GACAATAAAGTCTTAAACTGAA

**E4-Ins1-Kan_Rev**	AACACCACGCCCTGCGTCCCTGCTATGACTGGGTGATTTAAATAAATGTGGGCG-GACAAAATAGTTGG
**E4-Ins2_For**	TAAAGATTAAAGAGACGATGATTTTGAATTGATCAATAAA
**E4-Ins2_Rev**	CCTCCCCCAGCTTGAGGGTA
**E4-Ins2-Kan_For**	ATCCAGTAAAAAAAATAAAGATTAAAGAGACGATGATTTAAATGCGTGTGGGCG-GACAATAAAGTCTTAAACTGAA
**E4-Ins2-Kan_Rev**	CGTCAAGGTCCACCCCCTCCCCCAGCTTGAGGGTAATTTAAATAAATGTGGGCG-GACAAAATAGTTGG
**E4-Ins3_for**	TGATGACACCACCAGTGCATACTC
**E4-Ins3_rev**	GGCAGTGTGTTCTGGAGGGG
**E4-Ins3-Kan_For**	GGTACTGATGACACCACCAGTGCATACTCAATGTCATTTAAATCCGTGTGGGCG-GACAATAAAGTCTTAAACTGAA
**E4-Ins3-Kan_Rev**	ACATGGGCAGTGTGTTCTGGAGGGGACGCCACCTGATTTAAATAAATGTGGGCG-GACAAAATAGTTGG
**E4-Ins4_For**	TGCACTTCCTCAAACGCCCA
**E4-Ins4_Rev**	TGTGAGTTAATATGCAAATAAGGCG
**E4-Ins4-Kan_For**	ATACGTGCACTTCCTCAAACGCCCAAACTGGCGTCATTTAAATTCGTGTGGGCG-GACAATAAAGTCTTAAACTGAA
**E4-Ins4-Kan_Rev**	TTTTGTGTGAGTTAATATGCAAATAAGGCGTGAAAATTTAAATAAATGTGGGCG-GACAAAATAGTTGGGAACTGGGAG
**H3_B3-I0n_P1rev**	GGGTACCGAGCTCGAATTCACTGGCCGTCGAACACTCAG-CAGCTGACATAATTTTATTACATGAC
**H3_B3-I0n_P2rev**	CTTGCTGAAGTGAGTTTGGCGGATC
**H3_E4-I0n_P1rev**	GGGTACCGAGCTCGAATTCACTGGCCGTCGACCACGCAC-TTAAAGTCAACACATATTTTATTGCA
**H3_E4-I0n_P2rev**	CCTGCTGAAATGGGTTTCTTGACTCCAT
**H5_B3-I0n_P1for**	TGGTAGATTTATGTTTTTTTCTTGCGATTTTTTGTAGGTC
**H5_B3-I0n-P2for**	GGGGATCCTCTAGAGTCGACCTGCAGGCATTTATTACTTCTTGGGTGGGGACTT-GGATATATAA
**H5_E4-I0n_P1for**	TAGGGTGACTGGGTGATGACTGGTTTATT
**H5_E4-I0n_P2for**	GGGGATCCTCTAGAGTCGACCTGCAGGCATTTATGACTCAGGGGAGGGGACTTTG
**Ins_CMV-GLuc_Rev**	ATGCCTGCAGGTCGACTCTAGAGGATCCCCGAGTCGACAAGCTTGAATTCG
**Ins_mCMV-E4-1_for**	GAGCTCGGTACCCGGGGATCCTCTAGAGTCGTACTGAGTCATTAGGGACTTTCCAA
**Ins_mCMV-E4-1_rev**	GATTACGCCAAGCTTGCATGCCTGCAGGTCGAGTCGACAAGCTTGAATTCG
**Ins_mCMV-for**	CGACGGCCAGTGAATTCGAGCTCGGTACCCGTACTGAGTCATTAGGGACTTTCCAA
**PUC-AV_For**	AATTCTAGAGCTCGTCAGGTGGCAC
**PUC-AV_Rev**	TGCATCGCAGGAAAGAACATGTGAGC

Table S4. Overview of synthetic DNA used to construct sub-cloning vectors for application in this study.

Insert name	Sequence
**B3-Ins1**	TTTTGCTCACATGTTCTTTCCTGCGATGCAGAGTGGAAGCGCTTCTTTTGAGGGGGGAG-<br>TATTTAGCCCTTATCTGACGGGCAGGCTCCCACCATGGGCAGGAGTTCGTCAGAATGTCATGGGA<br>TCCACTGTGGATGGGAGACCCGTCCAGCCCGCCAATTCCTCAACGCTGACCTATGCCACTTT-<br>GAGTTCGTCACCATTGGATGCAGCTGCAGCCGCCGCCGCTACTGCTGCCGCCAACACCATCCTTG<br>GAATGGGCTATTATGGAAGCATCGTTGCCAATTCCAGTTCCTCTAA-<br>TAACCCTTCAACCCTGGCTGAGGACAAGCTACTTGTTCTCTTGGCGCAGCTCGAGGCCTTAACCC<br>AACGCTTAGGCGAACTGTCTAAGCAGGTGGCCCAGTTGCGTGAGCAAACTGAGTCTGCTGTT-<br>GCCACAGCAAAGTCTAAATAAAGATCTCAAATCAATAAATAAAGAAATACTTGTAAAACGACG<br>GCCAGTGAATTCGAGCTCGGTACCCGGGGATCCTCTAGAGTCGACCTGCAGGCATGCAA-<br>GCTTGGCGTAATCATGGTCATAGCTGTTTCCTGGATATAAAACAAATGAATGTTTATTTGATTTTT<br>CGCGCGCGGTATGCCCTGGACCATCGGTCTCGATCATTGAGAACGCGGTGGATCTTTTCCAG-<br>TACCCTGTAAAGGTGGGATTGAATGTTTAGATACATGGGCATTAGTCCGTCTCGGGGGTGGAGAT<br>AGCTCCATTGAAGAGCCTCTTGCTCCGGGGTAGTGTTATAAATCACCCAG-<br>TCATAGCAAGGTCGGAGTGCATGAATTCTAGAGCTCGTCAGGTGGCACTTTTC
**B3-Ins2**	TTTTGCTCACATGTTCTTTCCTGCGATGCAACTCTTAAAGAGTAGCCCTT-<br>GCCCGCGCTCATTTTGAAAACGGCGGGAATCACGTCACCCTTGGCACCTGTCCTTTGCCCTTGTC<br>ATGAGTAAAGAGATTCCCACGCCTTACATGTGGAGCTATCAGCCCCAAATGGGGTTGGCAG-<br>CAGGCGCTTCCCAGGACTACTCCACCCGCATGAATTGGCTTAGCGCCGGGCCCTCAATGATATC<br>ACGGGTTAATGATATACGAGCTTATCGAAACCAGTTACTCCTAGAACAGTCAGCTCTCAC-<br>CACCACACCCCGTCAACACCTTAATCCCCGAAATTGGCCCGCCACCCTGGTGTACCAGGAAAAT<br>CCCGCTCCCACCACCGTACTACTTCCTCGAGACGCCCAGGCCGAAGTTCAGATGACTAAC-<br>GCAGGTGTACAGCTGGCGGGCGGTTCCGCCCTATGTCGTCACCGACCTCAACAGAGTATAAAAC<br>GCCTGGTGATTAGAGGCCGAGGTATCCAGCTCAACGACGAGTCGGTTAGCTCTTCGCTT-<br>GGTCTGCGACCAGACGGAGTCTTCCAAATCGCCGGCTGTGGGAGATCTTCCTTCACTCCTCGTCA<br>GGCTGTGCTGACTTTGGAGAGTTCGTCCTCGCAGCCCCGCTCGGGCGGCATT-<br>GGAACTCTCCAGTTTGTGGAGGAGTTTACTCCCTCTGTCTACTTCAACCCCTTCTCCGGCTCTCCT<br>GGCCAGTACCCGGACGAGTTCATACCAAACTTCGACGCAATCAGCGAGTCAGTGGATGGC-<br>TATGATTGACGCGTCATTGCCCCTACCTTACCCAATCAAAATATTAATAAAGACACTGTAAAACG<br>ACGGCCAGTGAATTCGAGCTCGGTACCCGGGGATCCTCTAGAGTCGACCTGCAGGCATGCAA-<br>GCTTGGCGTAATCATGGTCATAGCTGTTTCCTGTACTTGAAATCAGCAATACAGTCTTTGTCAAA<br>ACTTTCTACCAGCAGCACCTCACCCTCTTCCCAACTCTGGTACTCTAAACGTCGGAGGGTGG-<br>CATACTTTCTCCACACTTTGAAAGGGATGTCAAATTTTATTTCCTCTTCTTTGCCCACAATCTTCAT<br>TTCTTTATCCCCAGATGGCCAAGCGAGCTCGGCTAAGCACTTCCTTCAACCCGGTG-<br>TACCCTTATGAAGATGAAAGCAGCTCACAACACCCATTTATAAATCCTGGTTTCATTTCCCCTGA<br>CGGGTTCACAAATTCTAGAGCTCGTCAGGTGGCACTTTTC
**B3-Ins3**	TTTTGCTCACATGTTCTTTCCTGCGATGCAAACTACTCAGGCAACCCTCATAAC-<br>CTCCCCATTTACCTTTTCCTATATTAGAGAAGATGACTGACAACAAAAATAAAGTTCAGTAAAAC<br>GACGGCCAGTGAATTCGAGCTCGGTACCCGGGGATCCTCTAGAGTCGACCTG-<br>CAGGCATGCAAGCTTGGCGTAATCATGGTCATAGCTGTTTCCTGACATTTTTTATTGAAATTCCTT<br>TTACAGTATTCGAGTAGTTATTTTGCCTCCCCCTTCCCATTTAACAGAATACAC-<br>CAATCTCTCCCCACGCACAGCTTTAAACATTTGGATACCATTAGAGATAGACATAGTTTTAGATT<br>CCACATTCCAAACAGTTTCAGAGCGAGCCAATCTGGGGTCAG-<br>TAATACATAAAAATGCATCGGGATAGTCTTTTAAAGCGCTTTCAAATTCTAGAGCTCGTCAGGTG<br>GCACTTTTC

**B3-Ins4**	TTTTGCTCACATGTTCTTTCCTGCGATGCAAAAAGGCACAGGAGAATAAAAAA-<br>TATAATTATTTCTCTGCTGCTGTTCAGGCAACGTTGCTCCCGGTCCCTCTAAATAGACATACAAAG<br>CCTCATCAGCCATGGCTTACCAGGCAAAGTACAGCGGGCGCACAAAGCACAAGCTCTAAA-<br>GAAGCTCTAAAAACACTCTCCAACCTCTCCACAATATATACACAAGCCCTAAACTGACGTAATG<br>GGAGTAAAGTGAAAAAAAAATACCGCCAAGCCCAACACACACCCCGAAACTGCGTCAGCAG-<br>GAAAAAGTACAGTTTCACTTCCGCATTCCCAACAAGCGTAACTTCCTCTTTCTCATGGTACGTCA<br>CATCCGATTAACTTGCGTAAAACGACGGCCAGTGAATTCGAGCTCGGTACCCGGG-<br>GATCCTCTAGAGTCGACCTGCAGGCATGCAAGCTTGGCGTAATCATGGTCATAGCTGTTTCCTGA<br>ACGTCATTTTCCCACGGTCGCGCCGCCCCTTTTAGCCGTTAACCCCGCAGCCAATCAC-<br>CACACAGCGCGCACTTTTTTAAATTACCTCATTTACATGTTGGCACCATTCCATAATTCTAGAGCT<br>CGTCAGGTGGCACTTTTC
**E4-Ins1**	TTTTGCTCACATGTTCTTTCCTGCGATGCATGGATGTGACGGAGGAC-<br>CTGCGACCCGATCATTTGGTGTTGTCCTGCACCGGGACGGAGTTCGGCTCCAGTGGGGAAGAATC<br>TGACTAGAGTGAGTAGTGTTTTGGGGAGGGAGAGGACCTGCATAAGGGGCAGAATGAT-<br>TAAAATCTGTGCTTTTCTGTGTGTTGCAGCAGCATGAGCGGAAACGGCTCCTTTGAGGGAGGGGT<br>ATTCAGCCCTTATCTGACGGGGCGTCTCCCCTCCTGGGCGGGAGTGCGTCAAAATGTGATGG-<br>GATCCACGGTGGACGGCCGGCCCGTACAGCCCGCGAACTCTTCAACCCTGACCTATGCAACCCT<br>GAGCTCCTCGTCGGTGGACGCAGCTGCCGCCG-<br>CAGCTGCTGCTTCTGCCGCCAGCGCCGTGCGCGGAATGGCCATGGGCGCCGGCTATTACGGCAC<br>TCTGGTGGCCAACTCGAGTTCCACTAATAATCCCGCCAGCCTGAACGAGGAGAA-<br>GCTGCTGCTGTTGATGGCCCAGCTCGAGGCCTTGACCCAGCGCCTGGGCGAGCTGACCCAGCAG<br>GTGGCTCAGCTGCAGGAGCAGACGCGGGCCGCGGTTGCCACGGTGAAATCCAAA-<br>TAAAAAATGAATCAATAAATAAACGGAGACGGGTAAAACGACGGCCAGTGAATTCGAGCTCGG<br>TACCCGGGGATCCTCTAGAGTCGACCTGCAGGCATGCAAGCTTGGCG-<br>TAATCATGGTCATAGCTGTTTCCTGTTGTTGATTTTAAAAATCAGAGTCTGAATCTTTATTTGATTT<br>TTCGCGCACGGTAGGCCCTGGACCACCGGCCTCGATCATTGAGCACCCGGTG-<br>GATCTTTTCCAAGACCCGGTAGAGGTGGGATTGGATATTGAGGTACATGGGCATGAGCCCGTCC<br>CGGGGGTGAAGGTAGCTCCATTGCAGGGCCTCGTGCTCGGGGGTGGTGTT-<br>GTAAATCACCCAGTCATAGCAGGGACGCAGGGCGTGAATTCTAGAGCTCGTCAGGTGGCACTTT<br>TC
**E4-Ins2**	TTTTGCTCACATGTTCTTTCCTGCGATGCAAAAAAATAAAGATTAAAGAGACGATGATTTT-<br>GAATTGATCAATAAAGAATGTAAAACGACGGCCAGTGAATTCGAGCTCGGTACCCGGGGATCCT<br>CTAGAGTCGACCTGCAGGCATGCAAGCTTGGCGTAATCATGGTCATAGCTGTTTCCTGCAC-<br>TTACTTGAAATCTGAAACCAGGTCTCTGTCCATGTTTTCTGTCAGCAGCACTTCGCTCCCCTCTTC<br>CCAGCTCTGGTACTGCAGGCCCCGGCGGGCTGCAAACTTCCTCCACACTCTGAAGGG-<br>GATGTCAAATTCCTCCTGTCCCTCAATCTTCATTTTTTATTTCTATTAGATGTCCAAAAAGCGCGC<br>GCGGGTGGATGATGGCTTCGACCCCGTGTATCCCTACGATGCAGACAACGCACCGAC-<br>CGTGCCCTTCATCAACCCTCCCTTCGTCTCTTCAGATGGATTCCAAGAAAAGCCCCTGGGGGTGT<br>TGTCCCTTAGGCTGGCCGACCCTGTCACCACCAAGAATGGGGAAATTACCCTCAAGCTGGGG-<br>GAGGGGGTGGACCTAATTCTAGAGCTCGTCAGGTGGCACTTTTC

**E4-Ins3**	TTTTGCTCACATGTTCTTTCCTGCGATGCATGATGACACCACCAGTGCAT-<br>ACTCAATGTCATTTTCATACACCTGGACTAACGGAAGCTATATCGGAGCAACATTTGGAGCTAAC<br>TCATACACCTTCTCCTACATAGCCCAACAATAATCCCACCCTGCATGCCAACCCAC-<br>CTTTTCCCTCTATTTATAAATGGAAACTGAAACAAAAATAAAGTTCGTAAAACGACGGCCAGTG<br>AATTCGAGCTCGGTACCCGGGGATCCTCTAGAGTCGACCTGCAGGCATGCAAGCTTGGCG-<br>TAATCATGGTCATAGCTGTTTCCTGAAGTGTTTTATTGATTCAACAGTTTTTCACAGGATTCGAGT<br>AGTTATTTTCCCTCCACCCTCCCATCTCATGGAATACACTATCCTCTCCCCAC-<br>GCACAGCCTTAAACATCTGAATGCTATTGGTAATGGACATGGTTTTGATCTCCACATTCCACACA<br>GTTTCAGAGCGAGACAGTCTCGGGTCGGTCAAGGAGATGAAACCCTCCGGGCACTCCTG-<br>CATCTGCACCTCACAGTTCAACAGCTGAGGGCTGTCCTCGGTGATTGGAATCACAGTTATCTGGA<br>ATAAGAGCGATGAGAATCATAATCCGCAAACGGGATCGGGCGGTTGTGGCG-<br>CATCAGGCCCCGCAGCAGTCGCTGTCTGCGCCGCTCCGTCAAGCTGCTACTCAAGGGGTCCGGG<br>TCCAGGGACTCCCTGCGCATGATGCCAATGGCCCTGAGCATCAGTCGCCTGGTAC-<br>GGCGGGCGCAGCAGCGGATGCGGATCTCACTCAGGTCGGAGCAGTACGTGCAGCACAGCACCA<br>CCAAGTTGTTCAACAGTCCATAGTTCAACGTGCTCCAGCCAAAACTCATTTGTGGAAC-<br>TATGCTGCCCACATGTCCATCGTACCAGATCCTGATGTAAATCAGGTGGCGTCCCCTCCAGAACA<br>CACTGCCAATTCTAGAGCTCGTCAGGTGGCACTTTTC
**E4-Ins4**	TTTTGCTCACATGTTCTTTCCTGCGATGCATGCACTTCCTCAAAC-<br>GCCCAAACTGGCGTCATTTCCGGTTTCCCACGCTACGTCACCTCTCAACGACTTTCAAATTCCGTC<br>GACCGTTAAACACATCAGTTAGTAAAACGACGGCCAGTGAATTCGAGCTCGGTACCCGGG-<br>GATCCTCTAGAGTCGACCTGCAGGCATGCAAGCTTGGCGTAATCATGGTCATAGCTGTTTCCTGC<br>CCCGCCCCTAACGAACGCCGCTGTCACAGCCAATCAGCGCGCCCCATCCCCAAATTTTCAC-<br>GCCTTATTTGCATATTAACTCACAAATTCTAGAGCTCGTCAGGTGGCACTTTTC

Table S5. Genomic insertion sites, demonstrated by 30 nt sequences directly adjacent to transgene insertion.

5' homologous region	Site	3' homologous region
GTAATAAAATTATGTCAGCTGCTGAGTGTT	**B3-I0n**	TTATTACTTCTTGGGTGGGGACTTGGATAT
GACTTTGACCGTTTACGTGGAGGTTTCGAT	**B3-I0**	TACCGTGTTTTTCACCTAAATTTCCGCGTA
GATCTCAAATCAATAAATAAAGAAATACTT	**B3-I1**	GATATAAAACAAATGAATGTTTATTTGATT
TTACCCAATCAAAATATTAATAAAGACACT	**B3-I2**	TACTTGAAATCAGCAATACAGTCTTTGTCA
GAAGATGACTGACAACAAAAATAAAGTTCA	**B3-I3**	ACATTTTTTATTGAAATTCCTTTTACAGTA
CTCATGGTACGTCACATCCGATTAACTTGC	**B3-I4**	AACGTCATTTTCCCACGGTCGCGCCGCCCC
TAAAATATGTGTTGACTTTAAGTGCGTGGT	**E4-I0n**	TTATGACTCAGGGGAGGGGACTTTGGGTAT
GACTTTGACCGTTTACGTGGGGGTTTCGAT	**E4-I0**	TACCGTGTTTTTCACCTAAAGTTCCGCGTA
AAAAATGAATCAATAAATAAACGGAGACGG	**E4-I1**	TTGTTGATTTTAAAAATCAGAGTCTGAATC
ACGATGATTTTGAATTGATCAATAAAGAAT	**E4-I2**	CACTTACTTGAAATCTGAAACCAGGTCTCT
TAAATGGAAACTGAAACAAAAATAAAGTTC	**E4-I3**	AAGTGTTTTATTGATTCAACAGTTTTTCAC
AATTCCGTCGACCGTTAAACACATCAGTTA	**E4-I4**	CCCCGCCCCTAACGAACGCCGCTGTCACAG

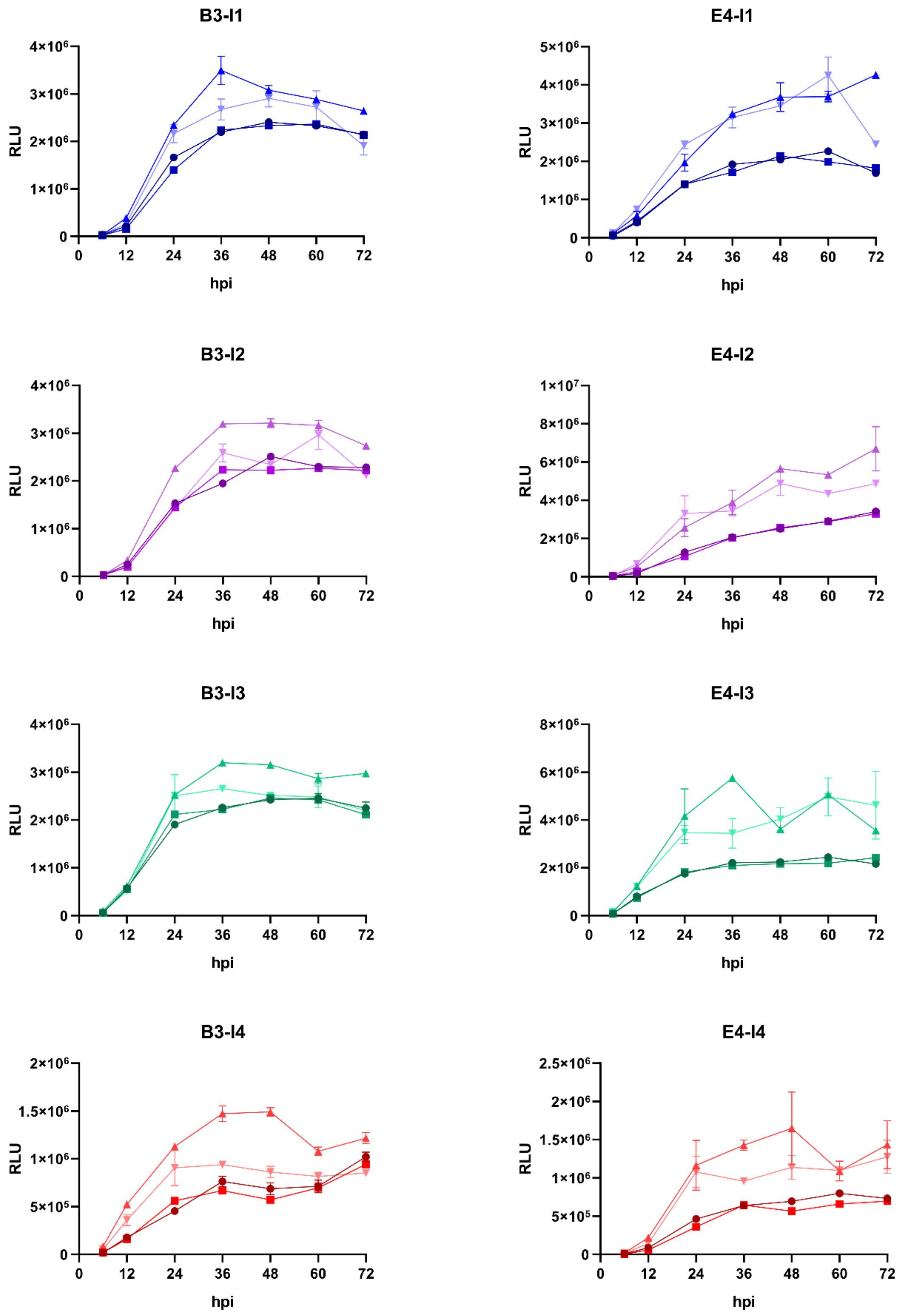

Figure S1. Expression profiles of insertion sites. Expression of transgene over time as determined by Luciferase assay for HAdV-B3 and HAdV-E4 based vectors. Colour and connecting lines indicate single experiments.
